# Reliability and Validity of the Chinese Version of FACIT-AI, a New Tool for Assessing Quality of Life in Patients with Malignant Ascites

**DOI:** 10.1089/jpm.2015.0061

**Published:** 2015-10-01

**Authors:** Yanni Lou, Linghui Lu, Yuan Li, Meng Liu, Jason M. Bredle, Liqun Jia

**Affiliations:** ^1^ Oncology Department of Integrative Medicine, China-Japan Friendship Hospital, Beijing, China.; ^2^ Beijing University of Chinese Medicine, Beijing, China.; ^3^ FACIT.org, Elmhurst, Illinois.

## Abstract

***Objective:*** The study objective was to determine the reliability and validity of the Chinese version of the Functional Assessment of Chronic Illness Therapy – Ascites Index (FACIT-AI).

***Methods:*** A forward-backward translation procedure was adopted to develop the Chinese version of the FACIT-AI, which was tested in 69 patients with malignant ascites. Cronbach's α, split-half reliability, and test-retest reliability were used to assess the reliability of the scale. The content validity index was used to assess the content validity, while factor analysis was used for construct validity and correlation analysis was used for criterion validity.

***Results:*** The Cronbach's α was 0.772 for the total scale, and the split-half reliability was 0.693. The test-retest correlation was 0.972. The content validity index for the scale was 0.8–1.0. Four factors were extracted by factor analysis, and these contributed 63.51% of the total variance. Item-total correlations ranged from 0.591 to 0.897, and these were correlated with visual analog scale scores (correlation coefficient, 0.889; *P*<0.01).

***Conclusions:*** The Chinese version of the FACIT-AI has good reliability and validity and can be used as a tool to measure quality of life in Chinese patients with malignant ascites.

## Introduction

Malignant ascites, a severe complication of advanced cancer, causes abdominal distension, anorexia, dyspnea, and eventually cachexia, thereby seriously reducing the patient's quality of life.^1^ The prognosis of malignant ascites is poor. The median survival ranges from several weeks to months, with an average survival of 20 weeks; the one-year survival rate is less than 10%.^2^ Palliative treatment of malignant ascites aims to reduce the volume of ascitic fluid and improve the patient's quality of life.^3^

At present there is no Chinese scale for the assessment of the quality of life of patients with malignant ascites. The Functional Assessment of Chronic Illness Therapy – Ascites Index (FACIT-AI), which was developed by Cella et al., is currently the only scale that aims to assess the quality of life of ascitic patients.^2^ The FACIT-AI has been proven to have favorable content validity and to be applicable to both clinical research and practice.^2^ The present study aims to introduce a Chinese version of this scale in order to provide a specific tool for quality-of-life assessments in ascitic patients. The reliability and validity of this version were determined.

## Methods

### Study subjects

We recruited patients with malignant tumors associated with ascites who had visited the China-Japan Friendship Hospital between March 2013 and March 2014. The inclusion criteria were as follows: (1) pathologically or cytologically confirmed malignant tumor and ultrasonography- or computed tomography-confirmed ascites, or cancer cells found in ascetic fluid at primary visit; (2) other ascites-associated clinical symptoms; (3) age >18 years; (4) education up to primary school or higher levels; and (5) voluntary participation in the study. The exclusion criteria were disorders affecting consciousness, hearing, or vision; extreme feebleness preventing the completion of the questionnaire; and refusal to participate in the study. A total of 69 patients meeting the above criteria were selected.

### Research tool

We used the FACIT-AI, which was collaboratively developed by five professors, including David Cella, at the Feinberg School of Medicine, Northwestern University, in 2013. The FACIT-AI includes a total of 13 items for the self-assessment of the health of the patient in the past seven days. In the Chinese version, each of these 13 items was scored using a five-point scale as follows: not at all, zero points; a little bit, one point; somewhat, two points; quite a bit, three points; and very much, four points. The total score was calculated by referring to the FACIT-AI scoring guidelines, version 4. The top 3 items were scored directly, while the next 10 items were reversed, i.e., the patient's response was subtracted from 4. The total score was obtained by summing up the direct and reversed scores. The higher the total score, the better the quality of life of the patient.

### Translation of FACIT-AI into Chinese

Under the authorization of the original authors, we carried out the procedures of translation, backward translation, cultural correction, and pretesting. First, two oncologists who were proficient in both English and Chinese independently translated the FACIT-AI into Chinese. A third translator compared the two versions and selected the best translation. If neither version was acceptable, another translation was carried out. Next, a qualified translator who had never read the original English version was required to back-translate the Chinese draft. Both the draft and the back-translation were sent to the original author for feedback, which was used to correct any unsatisfactory instances of translation. Next, five Chinese oncological experts with abundant clinical experience were invited to separately check the revised translation and correct the language from a cultural perspective as well as to evaluate the content validity. After this, 10 patients with malignant ascites filled in the Chinese version of the scale and were interviewed using another questionnaire whose contents included patients' understanding, relevance of items to disease conditions, usage of items displeasing to the patient, necessity of any new items, and evaluation of disputed items. Finally, the translation was again sent to the original author for another discussion to decide on the Chinese version of the FACIT-AI.

Patients who met the inclusion criteria were selected, and all investigators who would administer the survey were trained under the same conditions. After obtaining informed consent from both the patients and their relatives, we began the survey to collect general information and clinical data from the ascitic patients. All questionnaires had the same instructions and were filled in by the study subjects. During the survey completion process, the investigator was responsible for offering explanations of any unclear words and providing reading assistance to patients with a low level of education. The data thus collected were examined by the investigators, who also recorded the time taken for survey completion. Two days later, 20 patients were randomly chosen for a repeat survey.

### Statistical analysis

SPSS 17.0 (IBM, Armonk, NY) was used to analyze data. Cronbach's α and split-half reliability were used to assess internal consistency reliability, and Pearson correlation analysis and a paired t-test were used to assess test-retest reliability, wherein a coefficient of 0.65–0.70 indicated “acceptable” results, 0.70–0.80 indicated “fairly good” results, and 0.80–0.90 indicated “very good” results.

The content validity index (CVI) was used to assess content validity. A workshop was organized, and four chief oncologists and one chief nurse were invited to perform the assessments. The investigators explained the FACIT-AI and its assessment to the oncologists and nurse. Content validity was assessed using a four-point scale: 1, irrelevant; 2, weakly relevant; 3, moderately relevant; and 4, closely relevant. The item-level CVI (I-CVI) was calculated by dividing the appraiser total by the number of experts scoring 3 or 4 (reference range, 0.8–1.0). The scale-level CVI (S-CVI) included: (1) universal agreement (S-CVI/UA), namely, the percentage of items scored 3 or 4 by all experts from the total number of items (reference value, >0.80) and (2) average S-CVI (S-CVI/Ave), namely, the average value of I-CVI for the whole scale (reference value, >0.90).

Construct analysis was based on factor analysis. Both factor analysis and the maximum variance method were used for orthogonal rotation. The common factor was extracted using the standard of characteristic root >1 and a cumulative contribution ratio >40%. The factor load reflected the correlation intensity: the larger the factor load, the closer was the relationship between the item and the common factor (reference, absolute value >0.4).

Since the FACIT-AI is the only ascitic scale currently available, there is no gold standard for appropriate calibration. We therefore selected the Karnofsky performance status (KPS) scale and a visual analog scale (VAS) as the calibration criteria to assess the validity and reliability of the Chinese version of the FACIT-AI. Differences were considered significant at *P*<0.05 or *P*<0.01.

### Quality control during data collection

One researcher was designated to follow the entire survey in order to ensure data integrity. After survey completion, two staff members entered and checked the data simultaneously in order to maintain consistency between the entered data and the questionnaires.

## Results

### Translation of the FACIT-AI

Since most items of the FACIT-AI (except for BL2 and AI1) have been translated into Chinese during the translation of other scales and applied in clinical practice,^4–11^ they basically remain unchanged. The translation of BL2 was undisputed. Item AI1 (“I have been emotionally distressed”), however, only appears in the FACIT-AI, and its primary translation was not accepted by us. It was then translated to “I have been emotionally distressed” on the basis of the results of several workshops. Later, interviews of the 10 patients who underwent pretesting showed consistency between the patients' understanding and the original meaning, so this translation was adopted (see Table 1).

**Table 1.** Chinese Version of the FACIT-AI

*Item no.*	*Description*
C6	I have a good appetite
GF5	I am sleeping well
BMT5	I am able to get around by myself
B1	I have been short of breath
GP2	I have nausea
O2	I have been vomiting
ACT11	I have pain in my stomach area
O1	I have swelling in my stomach area
GP1	I have a lack of energy
ACT10	When I eat, I seem to get full quickly
BL2	I urinate more frequently than usual
Cx6	I am bothered by constipation
AI1	I have been emotionally distressed

### General data on the study subjects

We recruited a total of 69 patients with malignant ascites (including 10 patients for pretesting), among which there were 23 men and 46 women. Their ages ranged from 26 years to 88 years. The primary disease was gastric cancer in 16 patients; ovarian carcinoma, hepatoma, and intestinal cancer in 12 patients each; pancreatic carcinoma in 5 patients; lung cancer in 4 patients; breast cancer and peritoneal mesothelioma in 2 patients each; and Hodgkin's lymphoma in 1 patient. The primary disease was a tumor of unknown origin in 3 patients. The duration of ascites was 0.3–19 months, and the fluid depth was 2.6–13.8 cm. More information about the patients is summarized in Tables 2 and 3.

**Table 2.** Patient Characteristics

*Variable*	*All patients*
Age, median (range)	56.5 (26–88)
≤44 years, *n* (%)	19 (27.5)
45–59 years, *n* (%)	22 (31.9)
≥60 years, *n* (%)	28 (40.6)
Ethnicity, *n* (%)	
Han	69 (100)
Sex, *n* (%)	
Male	23 (33.3)
Female	46 (66.7)
Primary cancer, *n* (%)	
Gastric	16 (23.2)
Ovarian	12 (17.4)
Liver	12 (17.4)
Colorectal	12 (17.4)
Pancreatic	5 (7.2)
Lung	4 (5.8)
Breast	2 (2.9)
Others	6 (8.7)

**Table 3.** Duration of Ascites and Fluid Depth

	*Minimum*	*Maximum*	*Average*	*Median*
Duration of ascites (months)	0.3	19	3.43±4.06	3
Fluid depth (cm)	2.6	13.8	7.32±2.58	8

### Feasibility

The average time for completing the survey was 3.03±1.22 min. Most patients (89.9%, 62/69) thought that the items were easily understood, but 10.1% (7/69) patients, whose educational background was primary school, thought that the items were moderately difficult to understand. None of the patients believed the scale to be very difficult to understand.

### Reliability

#### Cronbach's α

The Chinese version of FACIT-AI had a total Cronbach's α of 0.772 and a Guttman split-half reliability of 0.693, suggesting good internal consistency.

#### Test–retest reliability

Among the 20 patients who took the survey twice, the Pearson correlation coefficient was 0.972 (*P*<0.01), and the result of a paired t-test was 1.222 (*P*=0.237, i.e., >0.05). Since no significant difference was observed between the test and the retest, we concluded that the Chinese version of the FACIT-AI had strong test-retest reliability and good stability.

### Validity

#### Content validity

Most items were scored a 3 or 4 by the five experts, except for BMT5 and O2, both of which were scored a 4. Using the formula recommended by Wan et al.,^7^ we calculated an I-CVI of 0.8–1.0. As for the S-CVI, universal agreement (S-CVI/UA) was 0.85 (reference range, ≥0.80), and the average S-CVI (S-CVI/Ave) was 0.97 (reference range, ≥0.90).

#### Construct validity

The Kaiser–Meyer–Olkin value was 0.637, and the result of the Bartlett test of sphericity was 296.52 (*P*<0.01). Since both met the significance level, factor analysis was applied for further examination. By the standard of characteristic root >1, 4 common factors were extracted to explain 29.76%, 13.30%, 11.65%, and 8.80% of the total variance, yielding a cumulative contribution of 63.51%. The factor load of the items was 0.591–0.887 (see Table 4).

**Table 4.** Load Values of the Four Common Factors in the Chinese Version of the FACIT-AI (*n*=69)

*Item No.*	*Factor 1*	*Factor 2*	*Factor 3*	*Factor 4*
C6	0.663	—	—	—
GF5	0.804	—	—	—
BMT5	0.677	—	—	—
B1	—	—	—	−0.736
GP2	—	0.897	—	—
02	—	0.885	—	—
ACT11	—	—	—	0.783
01	—	—	0.696	—
GP1	0.715	—	—	—
ACT10	0.591	—	—	—
BL2	—	—	0.775	—
Cx6	—	—	0.637	—
AI1	—	0.560	—	

—, an absolute factor load of <0.5.

#### Validity of calibration criteria

Both the KPS scores^12^ (determined by the researchers) and the VAS scores (assessed by the patients) were used as calibration criteria. FACIT-AI scores were significantly correlated with the VAS scores (*P*<0.01) but not with the KPS scores (*P*>0.05; see Table 5).

**Table 5.** Correlation of FACIT-AI Scores with KPS and VAS Scores

	*Calibration criterion*	*Correlation coefficient*	*P value*
FACIT-AI	KPS	−0.074	0.778
FACIT-AI	VAS	0.889	<0.001

FACIT-AI, Functional Assessment of Chronic Illness Therapy – Ascites Index; KPS, Karnofsky performance status; VAS, visual analog scale.

## Discussion

Improvement in quality of life is becoming increasingly important in evaluations of treatment effects,^13,14^ especially, in the case of diseases with poor prognosis due to complications like malignant ascites. At present, most quality-of-life studies in China have used scales such as the Quality of Life Questionnaire for Cancer Patients Version 3.0 (QLQ-C30)^15^ and the Nottingham Health Profile (NHP-QOL).^16^ However, these scales have some limitations when used to assess patients with malignant ascites: (1) too many items (the examples mentioned above have 58 and 38 items, respectively), which decreases patient acceptance; (2) missing data leading to difficult statistical analyses; and (3) overgeneralization of the scales, which may not be suitable for ascitic patients. In contrast, the FACIT-AI is the only quality-of-life scale specially designed for ascitic patients. The Chinese translation with its established reliability and validity will offer an effective tool for clinical practice and research.

Reliability reflects the stability of the measured results, and is most commonly assessed using methods such as internal consistency reliability, split-half reliability, and test-retest reliability. However, different studies have reported different reference ranges for reliability coefficients. In general, the minimum acceptable range is 0.65–0.70, while a moderately good range is 0.70–0.80 and a very good range is 0.80–0.90. In this study, the Cronbach's α coefficient was 0.772, suggesting moderately good internal consistency reliability, and the split-half coefficient was 0.693, indicating fine consistency between the two halves of the items of the scale. The test-retest reliability reflects consistency across time. The time interval before retesting varies between patient populations; cancer patients should be retested within one to two days after the primary test.^17^ The reference range for test-retest reliability is over 0.7 or 0.8,^18^ while our test-retest reliability was 0.972. Thus, no significant difference was found between the test and the retest, and the Chinese version of the FACIT-AI had moderately good test-retest reliability.

Validity refers to the degree to which a measurement tool can detect the actual value of a variable, and often includes content validity, construct validity, and criterion-related validity. After assessments by selected experts, the Chinese version of the FACIT-AI was found to have an I-CVI of 0.8–1.0 (reference, >0.78), an S-CVI/UA of 0.92 (reference, >0.8), and an S-CVI/Ave of 0.97 (reference, >0.9), indicating moderately good content validity.^7^ Construct validity is always described using explanatory factor analysis. In this study, a total of four common factors were extracted. Common factor one mainly reflected daily life. Common factor two was related to upper esophageal function. Common factor three mainly involved symptoms related to the volume of ascitic fluid, and common factor four reflected ascitic complications. The cumulative contribution of these factors to the total variance was 63.51% (reference, >40%), and all items had relatively high loads on the corresponding common factors and low loads on other factors, indicating that the Chinese version of the FACIT-AI had moderately good construct validity.^19^ As the first quality-of-life scale specially designed for ascitic patients, the FACIT-AI does not have any other scale as a calibration criterion for correlation. We used the objective KPS scale and the subjective VAS for correlation analysis, and found that the FACIT-AI was significantly correlated with VAS scores but not with KPS scores, which is consistent with the emphasis put by the World Health Organization on patients' subjective feelings.

The limitations of this study are the small sample size, inclusion of complex disease types, and the lack of similar reports about the reliability and validity of the FACIT-AI with which our results could be compared. Therefore, our investigation is only the first step towards the introduction of a Chinese version of the FACIT-AI. To validate this Chinese version of the FACIT-AI, more large-scale studies including diverse samples are required.

In summary, we developed a Chinese version of the FACIT-AI that has good practicability and moderately good reliability as well as validity. This version may be used for the assessment of quality of life and clinical efficacy in ascitic patients in China.
